# (*Z*)-3-(4-Methyl­phen­yl)-2-[(2-phenyl­cyclo­hex-2-en-1-yl)imino]-1,3-thia­zol­idin-4-one

**DOI:** 10.1107/S1600536812021691

**Published:** 2012-05-19

**Authors:** Chin Wei Ooi, Hoong-Kun Fun, Ching Kheng Quah, Murugan Sathishkumar, Alagusundaram Ponnuswamy

**Affiliations:** a X-ray Crystallography Unit, School of Physics, Universiti Sains Malaysia, 11800 USM, Penang, Malaysia; b Department of Organic Chemistry, School of Chemistry, Madurai Kamaraj University, Madurai-625 021, Tamil Nadu, India

## Abstract

The title compound, C_22_H_22_N_2_OS, exists in a *cis* conformation with respect to the N=C bond. The cyclo­hexene ring adopts a distorted sofa conformation. The thia­zolidine ring is essentially planar with a maximum deviation of 0.025 (2) Å and forms dihedral angles of 63.50 (7) and 57.52 (6)° with the benzene rings. In the crystal, mol­ecules are linked by C—H⋯O and C—H⋯N hydrogen bonds, generating *R*_2_^2^(8) ring motifs, and forming infinite chains along the *c* axis. The crystal is further consolidated by C—H⋯π inter­actions.

## Related literature

For details of thia­zolidin-4-one derivatives, see: Previtera *et al.* (1994 ▶); Sharma & Kumar (2000 ▶); Kato *et al.* (1999*a* ▶,*b* ▶); Tanabe *et al.* (1991 ▶); Rawal *et al.* (2005 ▶); Voss *et al.* (2003 ▶). For a related structure, see: Fun *et al.* (2011 ▶). For hydrogen-bond motifs, see: Bernstein *et al.* (1995 ▶). For ring conformations, see: Cremer & Pople (1975 ▶). For bond-length data, see: Allen *et al.* (1987 ▶). For stability of the temperature controller used in the data collection, see: Cosier & Glazer (1986 ▶).
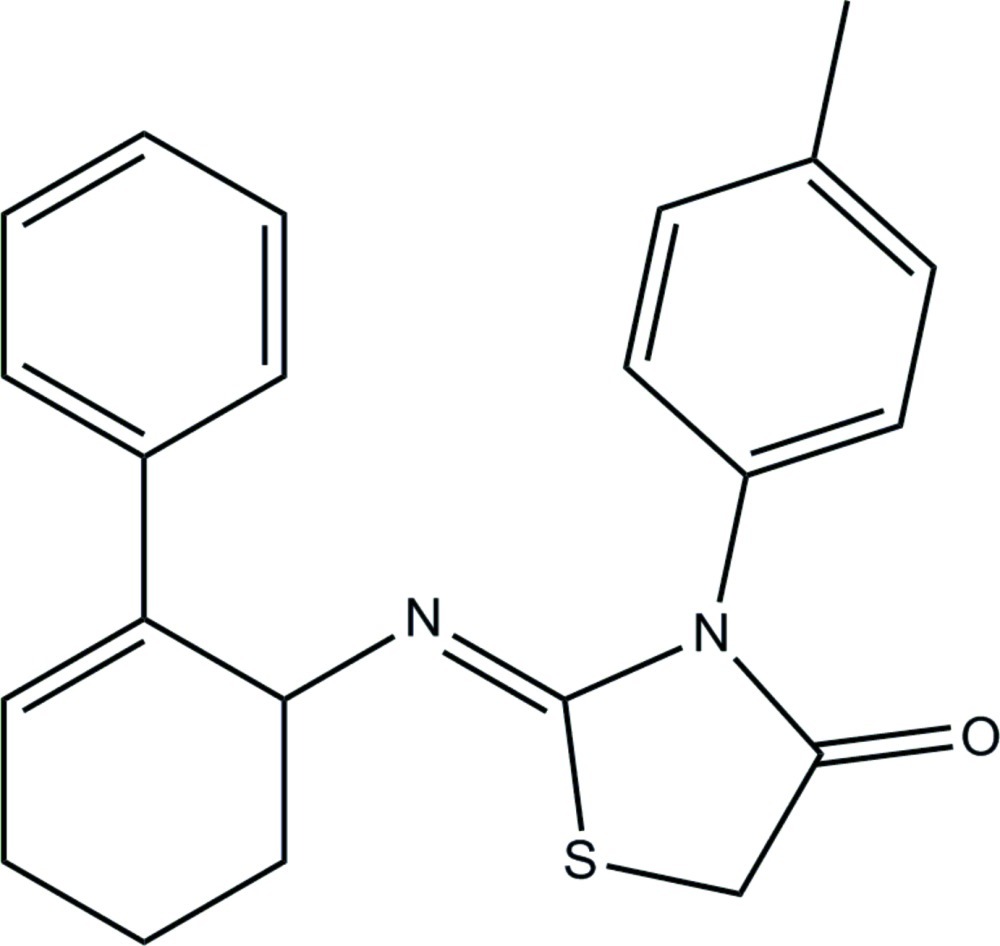


## Experimental

### 

#### Crystal data

C_22_H_22_N_2_OS
*M* *_r_* = 362.48
Monoclinic, 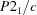

*a* = 9.2252 (4) Å
*b* = 17.6978 (8) Å
*c* = 12.8898 (4) Å
β = 119.289 (2)°
*V* = 1835.43 (13) Å^3^
*Z* = 4
Mo *K*α radiation
μ = 0.19 mm^−1^
*T* = 100 K
0.36 × 0.30 × 0.11 mm

#### Data collection

Bruker APEX DUO CCD area-detector diffractometer
Absorption correction: multi-scan (*SADABS*; Bruker, 2009 ▶) *T*_min_ = 0.935, *T*_max_ = 0.979
17337 measured reflections
5335 independent reflections
4496 reflections with *I* > 2σ(*I*)
*R*_int_ = 0.024

#### Refinement

*R*[*F* ^2^ > 2σ(*F* ^2^)] = 0.035
*wR*(*F* ^2^) = 0.100
*S* = 1.05
5335 reflections
236 parameters
H-atom parameters constrained
Δρ_max_ = 0.43 e Å^−3^
Δρ_min_ = −0.22 e Å^−3^

### 

Data collection: *APEX2* (Bruker, 2009 ▶); cell refinement: *SAINT* (Bruker, 2009 ▶); data reduction: *SAINT*; program(s) used to solve structure: *SHELXTL* (Sheldrick, 2008 ▶); program(s) used to refine structure: *SHELXTL*; molecular graphics: *SHELXTL*; software used to prepare material for publication: *SHELXTL* and *PLATON* (Spek, 2009 ▶).

## Supplementary Material

Crystal structure: contains datablock(s) global, I. DOI: 10.1107/S1600536812021691/bq2357sup1.cif

Structure factors: contains datablock(s) I. DOI: 10.1107/S1600536812021691/bq2357Isup2.hkl

Supplementary material file. DOI: 10.1107/S1600536812021691/bq2357Isup3.cml

Additional supplementary materials:  crystallographic information; 3D view; checkCIF report

## Figures and Tables

**Table 1:** Hydrogen-bond geometry (Å, °) *Cg*1 is the centroid of the C1–C6 benzene ring.

*D*—H⋯*A*	*D*—H	H⋯*A*	*D*⋯*A*	*D*—H⋯*A*
C11—H11*B*⋯O1^i^	0.97	2.35	3.2415 (14)	153
C14—H14*B*⋯N1^ii^	0.97	2.57	3.4020 (13)	143
C17—H17*A*⋯*Cg*1^iii^	0.93	2.86	3.6385 (14)	142

Symmetry codes: (i) 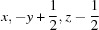; (ii) 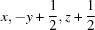; (iii) 
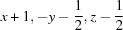
.

**Fig. 1.**
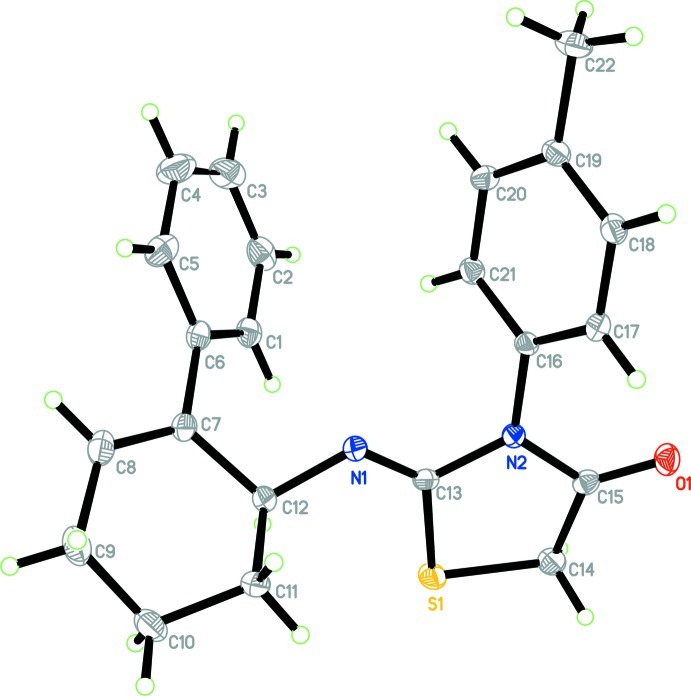
The molecular structure of the title compound, showing 50% probability displacement ellipsoids and the atom-numbering scheme.

**Fig. 2.**
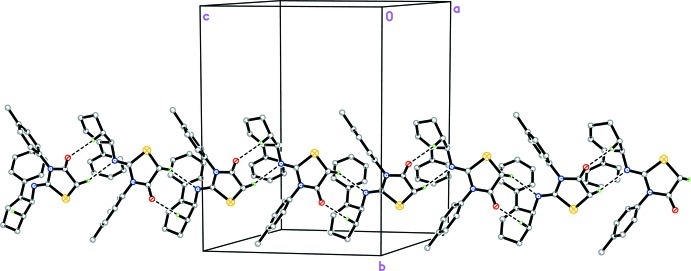
The crystal packing of the title compound, showing one-dimensional chains along the c axis. H atoms not involved in the intermolecular interactions (dashed lines) have been omitted for clarity.

## supplementary crystallographic information

### Comment

Thiazolidin-4-one derivatives are known to exhibit diverse bioactivities such as anti-histaminic (Previtera *et al.*, 1994), anti-microbial (Sharma & Kumar, 2000), (Kato *et al.*, 1999*a*), PAF antagonist (Tanabe *et al.*, 1991), cardioprotective (Kato *et al.*, 1999*b*), anti HIV (Rawal *et al.*, 2005), and tumor necrosis factor-α antagonist activities (Voss *et al.*, 2003).

The title compound (Fig. 1) exists in *cis* configuration with respect to the N1 ═C13 bond [N1 ═C13 = 1.2611 (13) Å]. The cyclohexene (C7–C12) ring adopts a distorted sofa conformation with atoms C10 and C11 deviating from the mean plane through the remaining atoms in opposite directions by -0.320 (2) Å and 0.331 (1) Å respectively. The puckering parameters are Q = 0.4995 (14) Å, θ = 129.75 (16)° and φ = 32.3 (2)° (Cremer & Pople, 1975). The thiazolidine (S1/N2/C13–C15) ring is essentially planar with a maximum deviation of 0.025 (2) Å at atom C14 and forms dihedral angles of 63.50 (7) ° and 57.52 (6) ° with the benzene rings (C1–C6 and C16–C21) respectively. The bond lengths (Allen *et al.*, 1987) and angles are within normal ranges and are comparable to the related structure (Fun *et al.*, 2011).

In the crystal structure (Fig. 2), molecules are linked by intermolecular C11—H11B···O1 and C14—H14B···N1 hydrogen bonds (Table 1), generating *R*_2_^2^ (8) ring motifs (Bernstein *et al.*, 1995) and forming infinite chains along the *c* axis. The crystal is further consolidated by C17—H17A···*Cg*1 interactions (Table 1), involving the centroid of the benzene ring (C1–C6; *Cg*1).

### Experimental

A mixture of 1-(2-phenylcyclohex-2-enyl)-3-(4-methyphenyl)thiourea (0.50 g, 2.3 mmol) and chloroacetyl chloride (0.35 g, 4.6 mmol) was heated to reflux in 1,4-dioxane (10 ml) at 100 °C for 5 h. The reaction mixture was washed with diluted sodium bicarbonate solution (25 ml) and dried over anhydrous sodium sulfate. The solvent was then evaporated under reduced pressure and the resulting residue was subjected to column chromatography using silica gel (60–120 mesh) as the stationary phase and petroleum ether-ethyl acetate (90:10) as the mobile phase to give the pure product. Yield: 0.64 g (77%); *M.p.*: 148–149 °C. Crystals suitable for X-ray study was obtained by recrystallization in dichloromethane.

### Refinement

All the H atoms were positioned geometrically and refined using a riding model with *U*_iso_(H) = 1.2 or 1.5*U*_eq_(C) (C—H = 0.93–0.98 Å). A rotating group model was applied to the methyl group.

### Crystal data

**Table:**

C_22_H_22_N_2_OS	*F*(000) = 768
*M**_r_* = 362.48	*D*_x_ = 1.312 Mg m^−^^3^
Monoclinic, *P*2_1_/*c*	Mo *K*α radiation, λ = 0.71073 Å
Hall symbol: -P 2ybc	Cell parameters from 7149 reflections
*a* = 9.2252 (4) Å	θ = 2.6–30.1°
*b* = 17.6978 (8) Å	µ = 0.19 mm^−^^1^
*c* = 12.8898 (4) Å	*T* = 100 K
β = 119.289 (2)°	Block, colorless
*V* = 1835.43 (13) Å^3^	0.36 × 0.30 × 0.11 mm
*Z* = 4	

### Data collection

**Table:**

Bruker APEX DUO CCD area-detector diffractometer	5335 independent reflections
Radiation source: fine-focus sealed tube	4496 reflections with *I* > 2σ(*I*)
Graphite monochromator	*R*_int_ = 0.024
φ and ω scans	θ_max_ = 30.1°, θ_min_ = 2.2°
Absorption correction: multi-scan (*SADABS*; Bruker, 2009)	*h* = −9→12
*T*_min_ = 0.935, *T*_max_ = 0.979	*k* = −24→24
17337 measured reflections	*l* = −18→18

### Refinement

**Table:**

Refinement on *F*^2^	Primary atom site location: structure-invariant direct methods
Least-squares matrix: full	Secondary atom site location: difference Fourier map
*R*[*F*^2^ > 2σ(*F*^2^)] = 0.035	Hydrogen site location: inferred from neighbouring sites
*wR*(*F*^2^) = 0.100	H-atom parameters constrained
*S* = 1.05	*w* = 1/[σ^2^(*F*_o_^2^) + (0.0506*P*)^2^ + 0.5577*P*] where *P* = (*F*_o_^2^ + 2*F*_c_^2^)/3
5335 reflections	(Δ/σ)_max_ < 0.001
236 parameters	Δρ_max_ = 0.43 e Å^−^^3^
0 restraints	Δρ_min_ = −0.22 e Å^−^^3^

### Special details

**Table:**

Experimental. The crystal was placed in the cold stream of an Oxford Cryosystems Cobra open-flow nitrogen cryostat (Cosier & Glazer, 1986) operating at 100 (1) K.
Geometry. All e.s.d.'s (except the e.s.d. in the dihedral angle between two l.s. planes) are estimated using the full covariance matrix. The cell e.s.d.'s are taken into account individually in the estimation of e.s.d.'s in distances, angles and torsion angles; correlations between e.s.d.'s in cell parameters are only used when they are defined by crystal symmetry. An approximate (isotropic) treatment of cell e.s.d.'s is used for estimating e.s.d.'s involving l.s. planes.
Refinement. Refinement of *F*^2^ against ALL reflections. The weighted *R*-factor *wR* and goodness of fit *S* are based on *F*^2^, conventional *R*-factors *R* are based on *F*, with *F* set to zero for negative *F*^2^. The threshold expression of *F*^2^ > σ(*F*^2^) is used only for calculating *R*-factors(gt) *etc*. and is not relevant to the choice of reflections for refinement. *R*-factors based on *F*^2^ are statistically about twice as large as those based on *F*, and *R*- factors based on ALL data will be even larger.

### Fractional atomic coordinates and isotropic or equivalent isotropic displacement parameters (Å^2^)

**Table:**

	*x*	*y*	*z*	*U*_iso_*/*U*_eq_	
S1	0.25977 (4)	0.358936 (15)	0.31318 (2)	0.01799 (8)	
O1	0.45923 (11)	0.16460 (5)	0.43965 (7)	0.01912 (17)	
N1	0.22548 (12)	0.30934 (5)	0.10025 (8)	0.01374 (18)	
N2	0.32300 (12)	0.22490 (5)	0.25893 (8)	0.01322 (17)	
C1	−0.13978 (15)	0.28972 (6)	−0.05611 (10)	0.0187 (2)	
H1A	−0.1117	0.3136	0.0155	0.022*	
C2	−0.25218 (15)	0.22988 (7)	−0.09409 (12)	0.0234 (2)	
H2A	−0.2979	0.2141	−0.0474	0.028*	
C3	−0.29663 (17)	0.19352 (8)	−0.20098 (13)	0.0287 (3)	
H3A	−0.3716	0.1536	−0.2260	0.034*	
C4	−0.22788 (18)	0.21747 (8)	−0.27017 (12)	0.0304 (3)	
H4A	−0.2581	0.1939	−0.3424	0.036*	
C5	−0.11379 (16)	0.27669 (7)	−0.23182 (11)	0.0244 (3)	
H5A	−0.0669	0.2915	−0.2781	0.029*	
C6	−0.06845 (14)	0.31438 (6)	−0.12433 (10)	0.0174 (2)	
C7	0.05345 (14)	0.37747 (6)	−0.08264 (10)	0.0163 (2)	
C8	0.06412 (16)	0.42580 (7)	−0.15897 (11)	0.0227 (2)	
H8A	−0.0077	0.4181	−0.2397	0.027*	
C9	0.18230 (18)	0.49102 (7)	−0.12470 (12)	0.0264 (3)	
H9A	0.1231	0.5352	−0.1704	0.032*	
H9B	0.2688	0.4792	−0.1442	0.032*	
C10	0.26201 (17)	0.50923 (6)	0.00802 (11)	0.0230 (2)	
H10A	0.3574	0.5419	0.0312	0.028*	
H10B	0.1827	0.5355	0.0239	0.028*	
C11	0.31672 (15)	0.43625 (6)	0.07990 (10)	0.0169 (2)	
H11A	0.3739	0.4482	0.1641	0.020*	
H11B	0.3939	0.4096	0.0622	0.020*	
C12	0.16769 (14)	0.38530 (6)	0.05117 (9)	0.0147 (2)	
H12A	0.1046	0.4068	0.0870	0.018*	
C13	0.26470 (13)	0.29626 (6)	0.20699 (9)	0.01284 (19)	
C14	0.34921 (16)	0.28935 (6)	0.43065 (10)	0.0177 (2)	
H14A	0.4507	0.3088	0.4968	0.021*	
H14B	0.2719	0.2776	0.4590	0.021*	
C15	0.38603 (14)	0.21927 (6)	0.38074 (9)	0.0145 (2)	
C16	0.31678 (14)	0.16074 (6)	0.18854 (9)	0.01263 (19)	
C17	0.45986 (14)	0.12024 (6)	0.21625 (9)	0.0150 (2)	
H17A	0.5622	0.1356	0.2781	0.018*	
C18	0.44769 (15)	0.05596 (6)	0.14971 (10)	0.0162 (2)	
H18A	0.5429	0.0281	0.1686	0.019*	
C19	0.29590 (15)	0.03267 (6)	0.05560 (10)	0.0158 (2)	
C20	0.15440 (15)	0.07559 (6)	0.02786 (10)	0.0161 (2)	
H20A	0.0526	0.0615	−0.0360	0.019*	
C21	0.16391 (14)	0.13906 (6)	0.09446 (10)	0.0147 (2)	
H21A	0.0688	0.1668	0.0762	0.018*	
C22	0.28358 (17)	−0.03726 (6)	−0.01526 (11)	0.0216 (2)	
H22A	0.3894	−0.0623	0.0201	0.032*	
H22B	0.2519	−0.0232	−0.0955	0.032*	
H22C	0.2016	−0.0707	−0.0154	0.032*	

### Atomic displacement parameters (Å^2^)

**Table:**

	*U*^11^	*U*^22^	*U*^33^	*U*^12^	*U*^13^	*U*^23^
S1	0.02608 (16)	0.01403 (13)	0.01548 (13)	0.00194 (11)	0.01144 (12)	−0.00189 (9)
O1	0.0227 (4)	0.0196 (4)	0.0144 (4)	0.0029 (3)	0.0086 (3)	0.0039 (3)
N1	0.0142 (4)	0.0123 (4)	0.0127 (4)	0.0011 (3)	0.0050 (4)	0.0001 (3)
N2	0.0160 (4)	0.0119 (4)	0.0107 (4)	0.0013 (4)	0.0057 (4)	0.0000 (3)
C1	0.0143 (5)	0.0179 (5)	0.0201 (5)	0.0045 (4)	0.0055 (4)	0.0026 (4)
C2	0.0146 (5)	0.0204 (5)	0.0317 (6)	0.0025 (5)	0.0087 (5)	0.0046 (5)
C3	0.0160 (6)	0.0230 (6)	0.0357 (7)	−0.0027 (5)	0.0040 (5)	−0.0039 (5)
C4	0.0246 (6)	0.0316 (7)	0.0247 (6)	−0.0042 (6)	0.0042 (5)	−0.0092 (5)
C5	0.0214 (6)	0.0286 (6)	0.0185 (5)	−0.0005 (5)	0.0063 (5)	−0.0023 (5)
C6	0.0135 (5)	0.0178 (5)	0.0159 (5)	0.0028 (4)	0.0033 (4)	0.0020 (4)
C7	0.0141 (5)	0.0160 (5)	0.0151 (5)	0.0027 (4)	0.0044 (4)	0.0016 (4)
C8	0.0225 (6)	0.0236 (5)	0.0170 (5)	0.0011 (5)	0.0057 (5)	0.0050 (4)
C9	0.0285 (7)	0.0219 (6)	0.0255 (6)	−0.0005 (5)	0.0107 (5)	0.0092 (5)
C10	0.0254 (6)	0.0137 (5)	0.0284 (6)	−0.0002 (5)	0.0121 (5)	0.0021 (4)
C11	0.0178 (5)	0.0136 (4)	0.0171 (5)	−0.0007 (4)	0.0067 (4)	−0.0015 (4)
C12	0.0168 (5)	0.0119 (4)	0.0137 (5)	0.0019 (4)	0.0060 (4)	0.0007 (4)
C13	0.0126 (5)	0.0118 (4)	0.0133 (4)	−0.0013 (4)	0.0057 (4)	−0.0022 (4)
C14	0.0244 (6)	0.0168 (5)	0.0141 (5)	0.0000 (5)	0.0110 (5)	−0.0011 (4)
C15	0.0153 (5)	0.0164 (5)	0.0122 (5)	−0.0022 (4)	0.0072 (4)	−0.0009 (4)
C16	0.0161 (5)	0.0108 (4)	0.0115 (4)	0.0001 (4)	0.0070 (4)	0.0001 (3)
C17	0.0145 (5)	0.0164 (5)	0.0118 (4)	0.0007 (4)	0.0047 (4)	0.0008 (4)
C18	0.0181 (5)	0.0154 (5)	0.0157 (5)	0.0035 (4)	0.0089 (4)	0.0020 (4)
C19	0.0214 (6)	0.0122 (4)	0.0154 (5)	−0.0004 (4)	0.0103 (5)	−0.0001 (4)
C20	0.0160 (5)	0.0151 (5)	0.0153 (5)	−0.0026 (4)	0.0061 (4)	−0.0018 (4)
C21	0.0137 (5)	0.0136 (4)	0.0161 (5)	0.0010 (4)	0.0067 (4)	0.0007 (4)
C22	0.0280 (6)	0.0150 (5)	0.0241 (6)	−0.0016 (5)	0.0146 (5)	−0.0052 (4)

### Geometric parameters (Å, º)

**Table:**

S1—C13	1.7796 (10)	C9—H9B	0.9700
S1—C14	1.8077 (11)	C10—C11	1.5244 (15)
O1—C15	1.2112 (13)	C10—H10A	0.9700
N1—C13	1.2611 (13)	C10—H10B	0.9700
N1—C12	1.4702 (13)	C11—C12	1.5305 (16)
N2—C15	1.3857 (13)	C11—H11A	0.9700
N2—C13	1.4072 (13)	C11—H11B	0.9700
N2—C16	1.4371 (12)	C12—H12A	0.9800
C1—C2	1.3928 (17)	C14—C15	1.5104 (14)
C1—C6	1.4010 (16)	C14—H14A	0.9700
C1—H1A	0.9300	C14—H14B	0.9700
C2—C3	1.3884 (19)	C16—C17	1.3865 (15)
C2—H2A	0.9300	C16—C21	1.3905 (15)
C3—C4	1.390 (2)	C17—C18	1.3957 (14)
C3—H3A	0.9300	C17—H17A	0.9300
C4—C5	1.3935 (19)	C18—C19	1.3929 (16)
C4—H4A	0.9300	C18—H18A	0.9300
C5—C6	1.4043 (16)	C19—C20	1.3971 (16)
C5—H5A	0.9300	C19—C22	1.5091 (15)
C6—C7	1.4863 (16)	C20—C21	1.3903 (14)
C7—C8	1.3431 (15)	C20—H20A	0.9300
C7—C12	1.5251 (15)	C21—H21A	0.9300
C8—C9	1.4983 (18)	C22—H22A	0.9600
C8—H8A	0.9300	C22—H22B	0.9600
C9—C10	1.5298 (18)	C22—H22C	0.9600
C9—H9A	0.9700		
			
C13—S1—C14	92.68 (5)	C12—C11—H11B	109.4
C13—N1—C12	119.17 (9)	H11A—C11—H11B	108.0
C15—N2—C13	117.04 (8)	N1—C12—C7	107.95 (8)
C15—N2—C16	121.57 (9)	N1—C12—C11	109.93 (9)
C13—N2—C16	121.39 (8)	C7—C12—C11	111.66 (9)
C2—C1—C6	120.91 (11)	N1—C12—H12A	109.1
C2—C1—H1A	119.5	C7—C12—H12A	109.1
C6—C1—H1A	119.5	C11—C12—H12A	109.1
C3—C2—C1	120.59 (12)	N1—C13—N2	121.51 (9)
C3—C2—H2A	119.7	N1—C13—S1	128.54 (8)
C1—C2—H2A	119.7	N2—C13—S1	109.94 (7)
C2—C3—C4	119.32 (12)	C15—C14—S1	107.95 (7)
C2—C3—H3A	120.3	C15—C14—H14A	110.1
C4—C3—H3A	120.3	S1—C14—H14A	110.1
C3—C4—C5	120.29 (12)	C15—C14—H14B	110.1
C3—C4—H4A	119.9	S1—C14—H14B	110.1
C5—C4—H4A	119.9	H14A—C14—H14B	108.4
C4—C5—C6	121.06 (12)	O1—C15—N2	124.34 (10)
C4—C5—H5A	119.5	O1—C15—C14	124.16 (9)
C6—C5—H5A	119.5	N2—C15—C14	111.49 (9)
C1—C6—C5	117.82 (11)	C17—C16—C21	120.91 (10)
C1—C6—C7	120.77 (10)	C17—C16—N2	120.46 (10)
C5—C6—C7	121.40 (10)	C21—C16—N2	118.61 (10)
C8—C7—C6	121.81 (11)	C16—C17—C18	118.87 (10)
C8—C7—C12	120.86 (11)	C16—C17—H17A	120.6
C6—C7—C12	117.33 (9)	C18—C17—H17A	120.6
C7—C8—C9	125.32 (11)	C19—C18—C17	121.38 (10)
C7—C8—H8A	117.3	C19—C18—H18A	119.3
C9—C8—H8A	117.3	C17—C18—H18A	119.3
C8—C9—C10	111.99 (10)	C18—C19—C20	118.51 (10)
C8—C9—H9A	109.2	C18—C19—C22	121.05 (10)
C10—C9—H9A	109.2	C20—C19—C22	120.44 (11)
C8—C9—H9B	109.2	C21—C20—C19	120.82 (11)
C10—C9—H9B	109.2	C21—C20—H20A	119.6
H9A—C9—H9B	107.9	C19—C20—H20A	119.6
C11—C10—C9	109.65 (10)	C20—C21—C16	119.48 (10)
C11—C10—H10A	109.7	C20—C21—H21A	120.3
C9—C10—H10A	109.7	C16—C21—H21A	120.3
C11—C10—H10B	109.7	C19—C22—H22A	109.5
C9—C10—H10B	109.7	C19—C22—H22B	109.5
H10A—C10—H10B	108.2	H22A—C22—H22B	109.5
C10—C11—C12	111.17 (10)	C19—C22—H22C	109.5
C10—C11—H11A	109.4	H22A—C22—H22C	109.5
C12—C11—H11A	109.4	H22B—C22—H22C	109.5
C10—C11—H11B	109.4		
			
C6—C1—C2—C3	−0.30 (18)	C15—N2—C13—N1	170.63 (10)
C1—C2—C3—C4	−0.06 (19)	C16—N2—C13—N1	−9.58 (16)
C2—C3—C4—C5	0.9 (2)	C15—N2—C13—S1	−8.88 (12)
C3—C4—C5—C6	−1.3 (2)	C16—N2—C13—S1	170.91 (8)
C2—C1—C6—C5	−0.13 (17)	C14—S1—C13—N1	−176.19 (11)
C2—C1—C6—C7	−179.04 (10)	C14—S1—C13—N2	3.28 (8)
C4—C5—C6—C1	0.94 (18)	C13—S1—C14—C15	2.27 (9)
C4—C5—C6—C7	179.84 (12)	C13—N2—C15—O1	−170.26 (10)
C1—C6—C7—C8	−147.19 (12)	C16—N2—C15—O1	9.95 (17)
C5—C6—C7—C8	33.94 (17)	C13—N2—C15—C14	10.80 (13)
C1—C6—C7—C12	33.08 (15)	C16—N2—C15—C14	−168.99 (10)
C5—C6—C7—C12	−145.79 (11)	S1—C14—C15—O1	173.60 (10)
C6—C7—C8—C9	179.62 (11)	S1—C14—C15—N2	−7.46 (12)
C12—C7—C8—C9	−0.66 (19)	C15—N2—C16—C17	−55.74 (14)
C7—C8—C9—C10	−14.03 (18)	C13—N2—C16—C17	124.48 (11)
C8—C9—C10—C11	44.52 (15)	C15—N2—C16—C21	122.51 (11)
C9—C10—C11—C12	−62.93 (13)	C13—N2—C16—C21	−57.26 (13)
C13—N1—C12—C7	−152.62 (10)	C21—C16—C17—C18	−1.47 (15)
C13—N1—C12—C11	85.36 (12)	N2—C16—C17—C18	176.74 (9)
C8—C7—C12—N1	−137.11 (11)	C16—C17—C18—C19	0.97 (15)
C6—C7—C12—N1	42.63 (12)	C17—C18—C19—C20	0.54 (15)
C8—C7—C12—C11	−16.16 (15)	C17—C18—C19—C22	−179.50 (10)
C6—C7—C12—C11	163.57 (9)	C18—C19—C20—C21	−1.59 (15)
C10—C11—C12—N1	167.43 (9)	C22—C19—C20—C21	178.45 (10)
C10—C11—C12—C7	47.65 (12)	C19—C20—C21—C16	1.12 (15)
C12—N1—C13—N2	−179.08 (10)	C17—C16—C21—C20	0.45 (15)
C12—N1—C13—S1	0.33 (16)	N2—C16—C21—C20	−177.80 (9)

### Hydrogen-bond geometry (Å, º)

Cg1 is the centroid of the C1–C6 benzene ring.

**Table:**

*D*—H···*A*	*D*—H	H···*A*	*D*···*A*	*D*—H···*A*
C11—H11*B*···O1^i^	0.97	2.35	3.2415 (14)	153
C14—H14*B*···N1^ii^	0.97	2.57	3.4020 (13)	143
C17—H17*A*···*Cg*1^iii^	0.93	2.86	3.6385 (14)	142

Symmetry codes: (i) *x*, −*y*+1/2, *z*−1/2; (ii) *x*, −*y*+1/2, *z*+1/2; (iii) *x*+1, −*y*−1/2, *z*−1/2.
